# PPGSynth: An Innovative Toolbox for Synthesizing Regular and Irregular Photoplethysmography Waveforms

**DOI:** 10.3389/fmed.2020.597774

**Published:** 2020-11-02

**Authors:** Qunfeng Tang, Zhencheng Chen, John Allen, Aymen Alian, Carlo Menon, Rabab Ward, Mohamed Elgendi

**Affiliations:** ^1^Department of Electrical and Computer Engineering, University of British Columbia, Vancouver, BC, Canada; ^2^School of Electronic Engineering and Automation, Guilin University of Electronic Technology, Guilin, China; ^3^Faculty of Medical Sciences, Population Health Sciences Institute, Newcastle University, Newcastle upon Tyne, United Kingdom; ^4^Research Centre for Intelligent Healthcare, Coventry University, Coventry, United Kingdom; ^5^Yale School of Medicine, Yale University, New Haven, CT, United States; ^6^School of Mechatronic Systems Engineering, Simon Fraser University, Burnaby, BC, Canada; ^7^Faculty of Medicine, University of British Columbia, Vancouver, BC, Canada; ^8^BC Children's & Women's Hospital, Vancouver, BC, Canada

**Keywords:** digital health, data modeling, data generation, big data, biosignal generation, PPG construction, signal simulation, generative model

## Abstract

Photoplethysmography (PPG) is increasingly used in digital health, exceptionally in smartwatches. The PPG signal contains valuable information about heart activity, and there is lots of research interest in its means and analysis for cardiovascular diseases. Unfortunately, to our knowledge, there is no arrhythmic PPG dataset publicly available—this paper attempt to provide a toolbox that can generate synthesized arrhythmic PPG signals. The model of a single PPG pulse in this toolbox utilizes two combined Gaussian functions. This toolbox supports synthesizing PPG waveform with regular heartbeats and three irregular heartbeats: compensation, interpolation, and reset. The user can generate a large amount of PPG data with a certain irregularity, with different sampling frequency, time length, and a range of noise types (Gaussian noise and multi-frequency noise) can be added to the synthesized PPG which can all be modified from the interface, and different types of arrhythmic PPGs (as calculated by the model) generated. The generation for large PPG datasets that simulate PPG collected from real humans could be used for testing the robustness of developed algorithms that are targeting arrhythmic PPG signals. Our PPG synthesis tool is publicly available.

## 1. Introduction

Photoplethysmogram (PPG) signal contains rich information about the cardiovascular system (1). In the past decade, some studies have used PPG to calculate heart rate, oxygen saturation, blood pressure, cardiac output, cardiac index, peripheral vascular resistance, and other indicators of cardiovascular function, and many algorithms were developed to calculate these indices (2).

Four PPG databases, at time of writing, are publicly available: Multiparameter Intelligent Monitoring in Intensive Care (MIMIC) (3), the University of Queensland Vital Signs Dataset (4), Vortal Dataset (5), and PPG-BP (6). The sampling frequency and time length of PPG signals are different in different databases; however, most algorithms designed for these databases are signal-independent. Additionally, it is still a challenge to evaluate the performance of these algorithms under different PPG types and different signal-to-noise ratios (SNR).

PPGSynth is developed to generate PPG signals across a wider range of sampling frequencies and time lengths. Three types of irregular PPG signals also can be generated by the PPGSynth tool. It can also conveniently manage parameters and graphical output through a graphical user interface (GUI). This toolbox does not require highly experienced users, but it is recommended that you have basic knowledge of PPG signal and cardiac irregularities.

## 2. Heartbeat Classification

The amplitude, duration, and waveform shape of PPG pulses tend to vary between persons, and they even differ from moment to moment in the same person. Premature heartbeats are typical irregular PPG beats. There are two different types of premature heartbeats, premature atrial contractions and premature ventricular contractions. This study only focuses on irregular PPG signals that have premature atrial contractions. Premature atrial contraction changes the waveform of PPG for two consecutive beats. In this study, these two beats are defined as the premature group, and the first beat of the premature group and the second beat of the premature group are defined as the first beat and second beat, respectively. The beats without the influence of premature contractions are defined as reference beats. The first beat duration is always less than the reference beat duration. Based on the difference between the durations of the first beat and second beat, Roskamm and Csapo (7), classified heartbeats into four types: compensation, reset, interpolation, and re-entry. Based on their analysis, these four types are defined as follows:

- Compensation: the second beat is prolonged, and the sum of the first beat duration and second beat duration is equal to the duration of two reference beats.
- Reset: the second beat is prolonged, but the sum of the first beat duration and second beat duration is less than the duration of two reference beats.
- Interpolation: the sum of the first beat duration and second beat duration is equal to one reference beat duration.
- Re-entry: the sum of the first beat duration and second beat duration is less than one reference beat duration. We could not find a template, within the four databases mentioned above, that satisfies the definition of re-entry. Therefore, the re-entry is not included in the current analysis.

**Figure 1:**
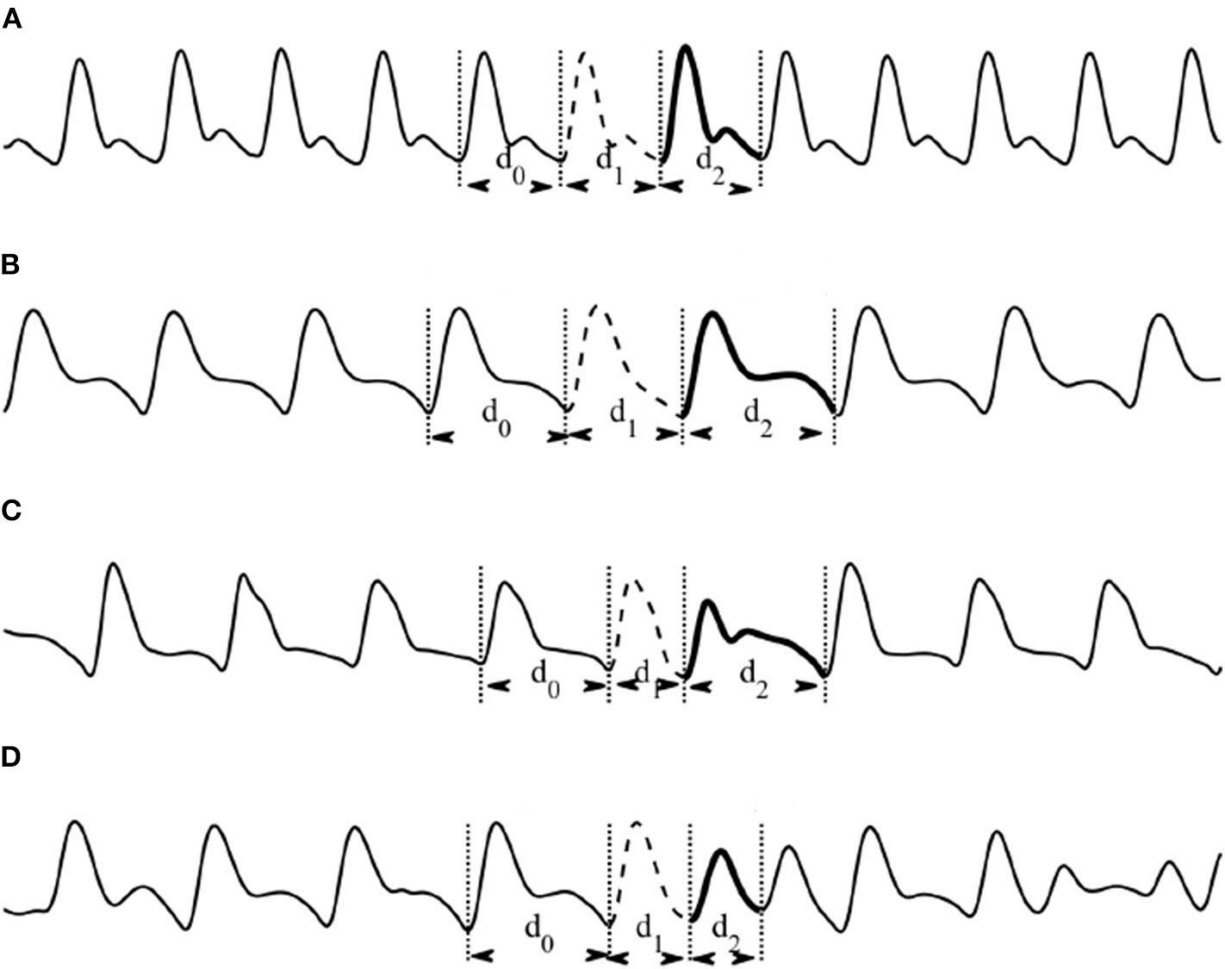
Types of heartbeat classification based sinus node response to atrial premature depolarization [adapted from (7, 8)]. **(A)** Regular: d_0_ = d_1_ = d_2_. **(B)** Compensation: d_1_ + d_2_ = 2d_0_. **(C)** Reset: d_0_ < d_1_ + d_2_ < 2d_0_. **(D)** Interpolation: d_1_ + d_2_ = d_0_.

A previous attempt (8) on the use of heartbeat classification using ECG signals inspired the classification of heartbeats in PPG signals. Based on the previous heartbeat classification (8), Figure 1A shows the regular heartbeats where the first beat, second beat, and third beat have equal durations (e.g., *d*_0_ = *d*_1_ = *d*_2_ = 1, 000 ms). On the other hand, Figure 1B shows the compensation phase, the second beat (e.g., *d*_1_ = 850 ms) is followed by a prolonged beat (e.g., *d*_2_ = 1150 ms) to compensate the two beats duration of 2,000 ms. During the reset phase (Figure 1C), the second beat (e.g., *d*_1_ = 650 ms) is followed by a prolonged beat (e.g., 1,150 ms), while in the interpolation (Figure 1D), the second beat (e.g., *d*_1_ = 400 ms) is followed by an irregular beat (e.g., *d*_2_ = 600 ms).

## 3. Methodology

The PPGSynth consists of three main parts: the model of a single PPG pulse, the pulse duration generator, and the noise generator.

### 3.1. Model of Single PPG Pulse

The single PPG pulse step is based on a recently published model (9) that simulates fingertip PPG waveforms. Note that the adopted model (9) is an early work on healthy subjects; however, this paper is about arrhythmic PPG beats relating to cardiovascular patient simulated recordings, which is definitely a new concept.

**Figure 2:**
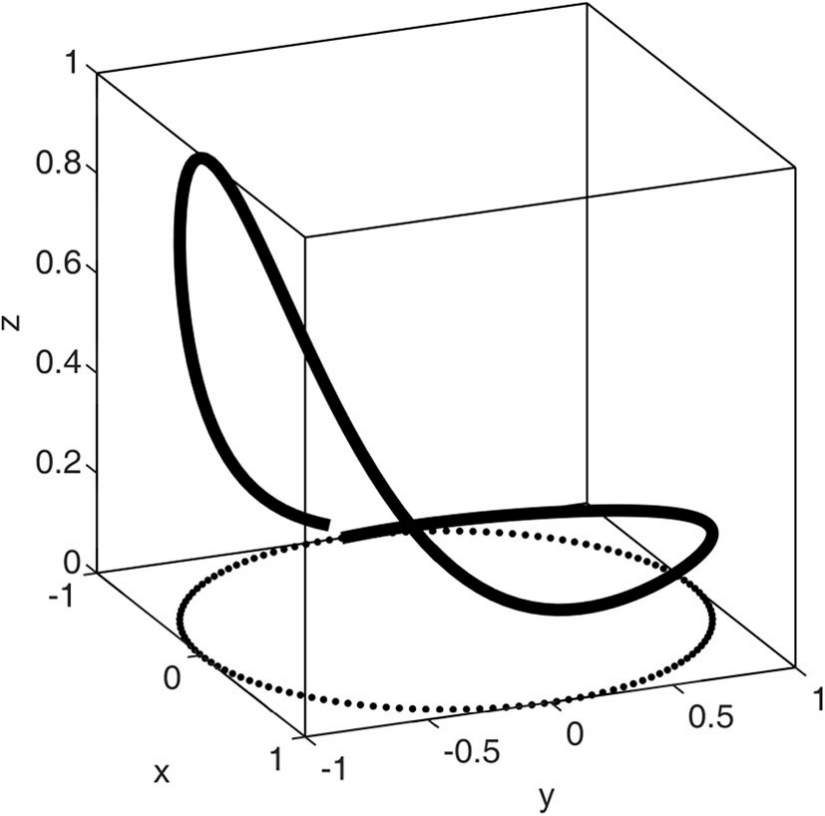
Motion trajectory of a single synthesized PPG waveform. This figure shows how the Gaussian model simulates a heartbeat of a PPG waveform.

The construction of a PPG waveform is regarded as a motion trajectory in the three-dimensional space established by the coordinate system (*x, y, z*). As shown in Figure 2, the periodicity of PPG is represented by a circular motion.

The motion trajectory in the (*x, y*) plane is the unit circle. One cycle of movement on the circle corresponds to a peak-to-peak interval or heartbeat. The trajectory in the *z* direction is the PPG signal. The systolic wave and diastolic wave are simulated in Gaussian functions. The equation of (*x, y, z*) is defined as follows:

(1) $$\{\begin{array}{l} x(t)=\cos\;(\omega (t-t_{0})-\pi )\\ y(t)=\sin\;(\omega (t-t_{0})-\pi )\\ z(t)=\sum_{i=1}^{2}a_{i}\exp(-\frac{{\left(\theta (t)-\theta_{i}\right)}^{2}}{2b_{i}^{2}}), \end{array}$$

where *t* is time, ω is the angular velocity (which is used to control the pulse duration), *t*_0_ is the end time of the previous beat,π is used to align the initial point of this model to the position of the onset in a PPG waveform, and *a*_*i*_, θ_*i*_, and *b*_*i*_ are the amplitude of the peak, the position of the center of the peak, and the standard deviation of Gaussian functions, respectively. Additionally, ω is calculated by:

(2) $$\begin{array}{l} \omega =\frac{2\pi }{T}, \end{array}$$

where *T* is the PPG pulse duration. θ is the four-quadrant inverse tangent of (*x, y*), which is introduced as an independent variable for motion in the *z* direction and is defined as:

(3) $$\begin{array}{l} \theta \left(t\right)=atan2\left(y\left(t\right),x\left(t\right)\right), \end{array}$$

with the changes to (*x, y*), θ is in the range of (−π, π).

The corresponding changes to *x*, *y*, and *z* over a single period are shown in Figure 2; these are repeated in the next pulses. In this figure, the pulse duration was 1 s, and the sampling frequency was 125 Hz. Obtaining a waveform of the synthetic PPG pulse that is close as possible to the real PPG pulse through calculation of model parameters is an optimization problem (finding the optimal parameters). The objective function was expressed as follows:

(4) $$\begin{array}{l} p^{*}=\arg\underset{p}{\min}\left(\overset{l}{\underset{n=1}{\sum }}{\left(z_{p}\left(n\right)-s\left(n\right)\right)}^{2}+\left(1-corr\left(z_{p}\left(n\right),s\left(n\right)\right)\right)\right), \end{array}$$

such that *p* = {*a*_1_, θ_1_, *b*_1_, *a*_2_, θ_2_, *b*_2_},

with the constraints:

(5) $$\{\begin{array}{l} 0\leq a_{2}<a_{1}\leq 1\\ 0\leq b_{1}<b_{2}\leq 3\\ -\pi \leq \theta_{2}<\theta_{1}\leq \pi , \end{array}$$

where *z*_*p*_(*n*) is the synthetic PPG, *l* is the length of the real PPG *s*(*n*), and *corr* is Pearson's linear correlation coefficient.

In this study, the interior-point (10) method was used to solve the optimization problem.

### 3.2. Variability of Parameters

In real-world PPG, the waveform often varies between pulses—sometimes dramatically so. To make the synthesized PPG closer to a real PPG, we used a Gaussian distribution to generate random parameters for our model. In this paper, the mean value and standard deviation of each parameter's Gaussian distribution are derived from real PPG signals, set by modeling a PPG pulse (from start of a pulse to the start of the consecutive pulse). For a PPG trace which has regular beats; we modeled all pulses in a 5-min PPG from the MIMIC database (3). However, for a PPG trace which has irregular beats, the waveform of the first beat and second beat in the premature group is distinct from the reference beat. We model three compensation segments (include the first beat and second beat) from one record of the Queensland database (4) to get the distribution of parameters in the compensation type. For reset, four reset segments from one record of the MIMIC database are used to get the distribution. For interpolation, three interpolation segments in one record from the Queensland database are used to calculate the parameters. The mean and standard deviation of these parameters are shown in Table 1. Note that since we do not have a high-quality re-entry PPG in our database, this toolbox does not support generating re-entry PPGs. To not change the irregular category, the duration ratio of irregular beat and regular beat in synthetic PPG uses a fixed value instead of a random number obeying Gaussian distribution. These fixed values are the mean of the ratio of the pulse duration of the irregular beat and regular beat in Table 1.

**Table 1:** The optimal parameters for each PPG template.

**PPG type**	**Beat**	***a*_1_**	***a*_2_**	***b*_1_**	***b*_2_**	**θ_1_**	**θ_2_**	**Ratio of pulse duration**
Regular	-	0.997 ± 0.028	0.225 ± 0.030	0.641 ± 0.034	0.937 ± 0.161	−1.471 ± 0.147	1.019 ± 0.102	-
Compensation	1*stBeat*	0.829 ± 0.010	0.420 ± 0.018	0.732 ± 0.033	1.219 ± 0.021	−1.008 ± 0.147	0.450 ± 0.167	0.830 ± 0.061
Compensation	2*ndBeat*	0.785 ± 0.034	0.405 ± 0.049	0.678 ± 0.036	1.115 ± 0.065	−1.792 ± 0.080	−0.607 ± 0.107	1.170 ± 0.061
Reset	1*stBeat*	0.774 ± 0.012	0.774 ± 0.012	0.647 ± 0.041	1.007 ± 0.046	−1.378 ± 0.180	0.173 ± 0.180	0.607 ± 0.019
Reset	2*ndBeat*	0.995 ± 0.002	0.197 ± 0.024	0.778 ± 0.055	1.045 ± 0.341	−1.809 ± 0.203	0.892 ± 0.325	0.596 ± 0.484
Interpolation	1*stBeat*	0.668 ± 0.151	0.490 ± 0.006	0.893 ± 0.034	1.428 ± 0.062	−0.627 ± 0.292	0.442 ± 0.635	0.561 ± 0.028
Interpolation	2*ndBeat*	0.595 ± 0.084	0.537 ± 0.092	0.889 ± 0.170	1.321 ± 0.289	−1.049 ± 0.207	−0.289 ± 0.480	0.475 ± 0.028

*Here, θ_1_ and θ_2_ are the locations of the first Gaussian distribution and the second Gaussian distribution, respectively. Here, a_1_ and a_2_ are the amplitudes, and, b_1_ and b_2_ are the widths of the first and second Gaussian distributions, respectively. Note that in the regular PPG heartbeat, all beats have similar durations based on the standard deviation*.

### 3.3. Pulse Duration

In this study, the PPG pulse duration is defined as the valley-to-valley interval. To generate a sequence of PPGs, a series of PPG pulse durations were needed. In this toolbox, reference pulse durations are generated based on the basic heart rate and signal time lengths, and then the reference pulse durations are randomly replaced by two consecutive irregular beats. The ratios of the first beat duration and second beat duration to the reference beat duration are calculated from each type of PPG templates, and the results are shown in Table 1.

### 3.4. Adding Noise

Two types of noise are available in this toolbox: white Gaussian noise and multi-frequency noise. Multi-frequency noise is a set of noises that have different amplitudes and frequencies. Each noise is generated as follows:

(6) $$\begin{array}{l} n\left(t\right)=A\sin2\pi ft, \end{array}$$

where *A* is the amplitude of the peak of the noise and *f* is the frequency of the noise.

If necessary, users can add one or more different amplitudes and frequency noises to the clean synthetic PPG signals.

## 4. Cubic Interpolation

The variability of parameters will make the endpoint value of one beat differ from the next beat's onset value. In this paper, cubic interpolation was used to smooth the synthetic PPG. Cubic spline interpolation involves a spline where each piece is a third-degree polynomial specified by its values and first derivatives at the corresponding domain interval's endpoints. The interpolation involved a total of 0.2 s around the onset. The previous sampling points of 0.05 s and the last sampling points of 0.05 s were used to fit the interpolation function. The middle 0.1-s samples' value is replaced by the corresponding samples' value generated by the interpolation function.

## 5. The Graphical User Interface

**Figure 3:**
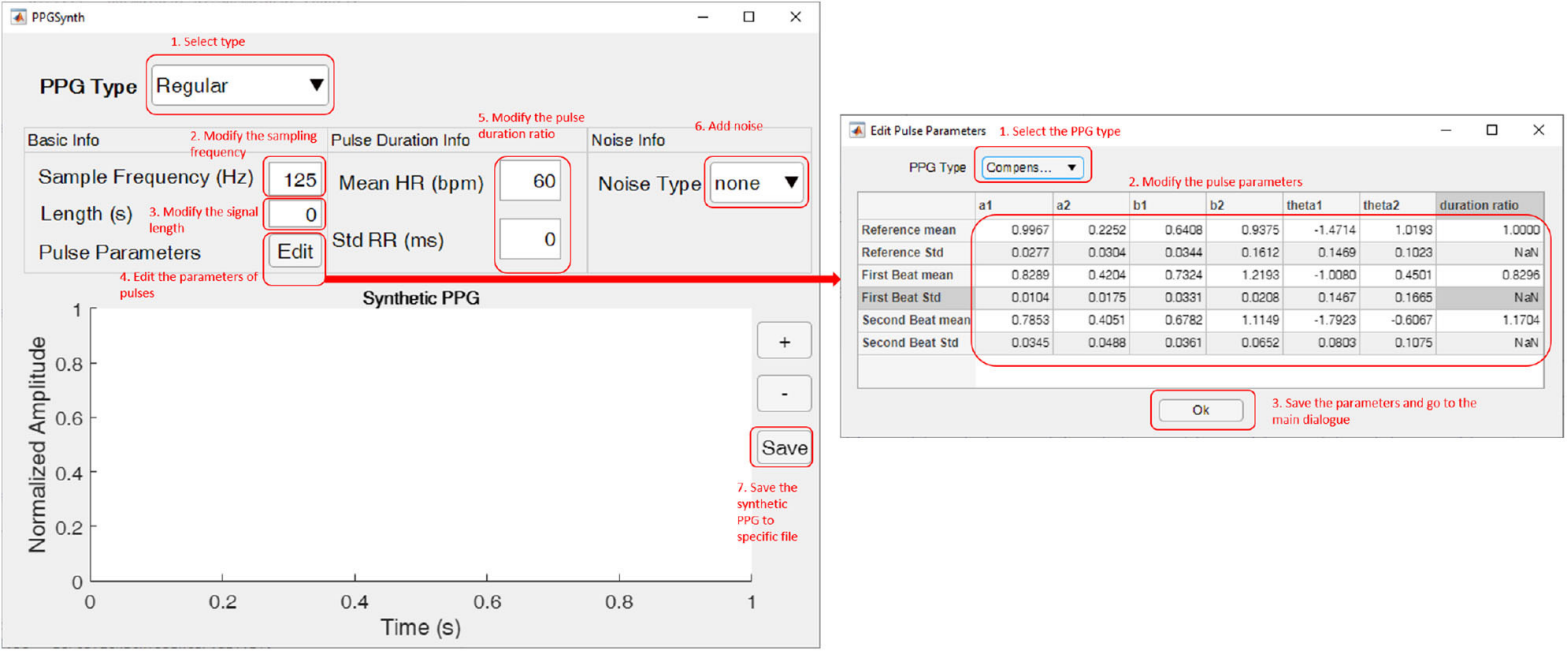
The graphical interface of PPGSynth. The left side is the main dialogue. The edited parameters dialogue on the right side will pop up after pressing the “Edit” button. The red text show the numbered steps for generating PPGs. This figure shows how to use the graphical interface of PPGSynth.

Figure 3 shows the main dialogue of the GUI. The first step is to select the type of synthetic PPG using the drop-down button in the upper left corner. Available options are regular or three types of irregular PPG. Then we can modify the sampling frequency and signal length in the “Basic Info” panel. Once we change any data, the GUI will attempt to generate the synthetic PPG and show it at the bottom of the dialogue.

By pressing the “Edit” button, users can modify the parameters of pulses in the pop-up dialogue. For a regular PPG, this toolbox uses the same parameters for different pulses. But for irregular PPGs, parameters are different in the first beat of the premature group, the second beat of the premature group, and the reference beat. Users can also modify the ratio of first beat duration and second beat duration to reference beat duration. The default value is shown in Table 1. Once done with editing pulse parameters, press the “OK” button to save these parameters and go to the main dialogue.

After setting the basic info, users should set some parameters to generate the pulse duration. For a regular PPG, users can modify the mean heart rate and standard deviation of the RR intervals in the “Pulse Duration Info” panel. For irregular PPG types, this panel changes to an “Irregular Duration Info” panel, where users can modify the basic heart rate and irregular times of the synthetic PPG. The basic heart rate and mean heart rate are in the range of 50 to 180. A warning dialogue pops up when the “Irregular Times” value is too large or too small relative the signal length. In this case, users should either decrease the irregular times or increase the length of the signals.

**Figure 4:**
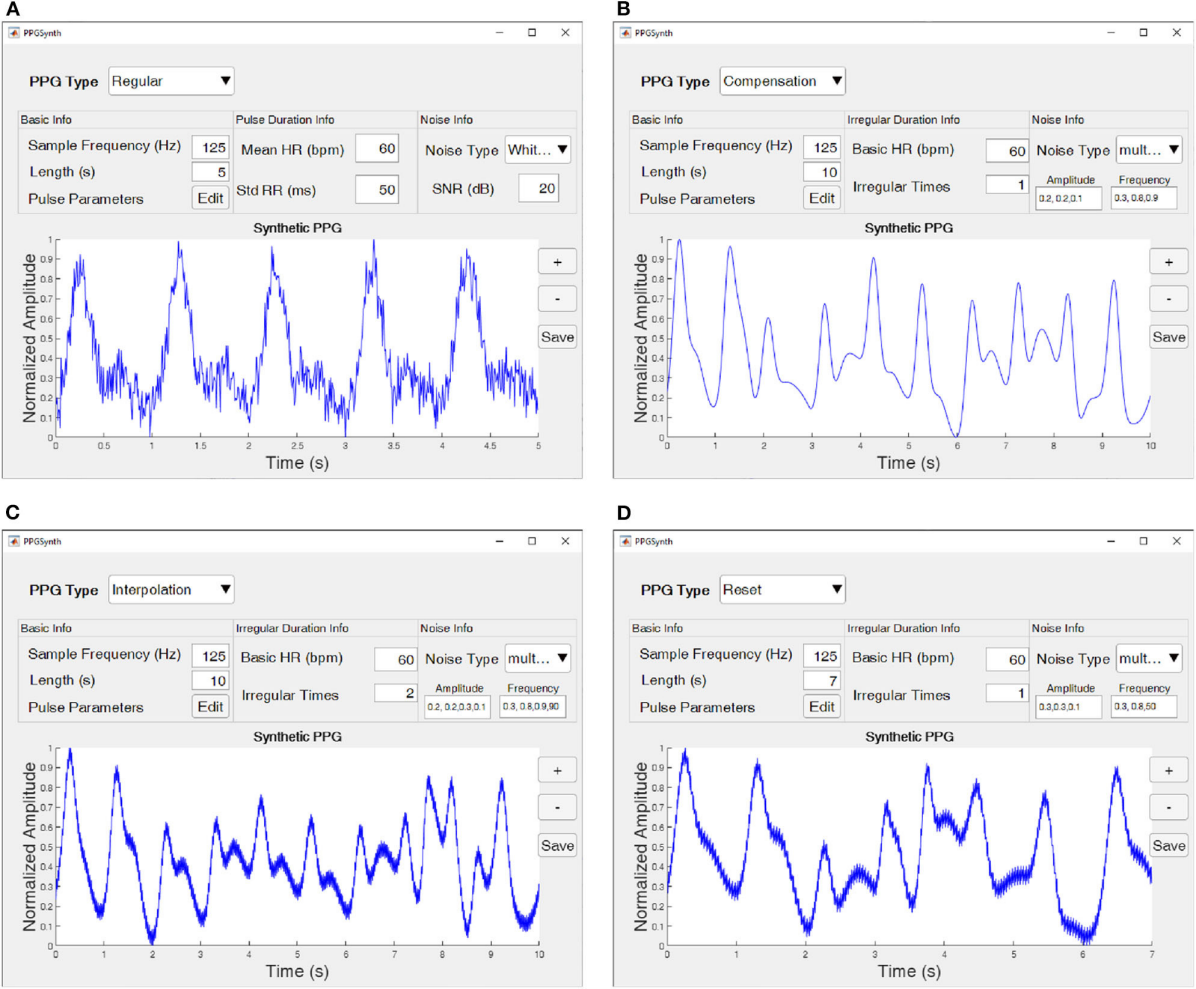
Four types of PPG heartbeat generated using the PPGSynth. **(A)** A 5-s regular PPG with white Gaussian noise. **(B)** A 10 s compensation PPG with multi-frequency noise. **(C)** A 10-s interpolation PPG with multi-frequency noise. **(D)** A 10-s reset PPG with multi-frequency noise. The parameters of each type shown at each subfigure. Additionally, the pulse parameters associated with each type are shown in Table 1.

If necessary, users can add noise to synthetic PPG. Two types of noise are available in the “Noise Info” panel: White Gaussian noise and multi-frequency noise. For white Gaussian noise, users can modify the signal-to-noise ratio (SNR). A 5-s regular PPG with white Gaussian noise is shown in Figure 4A. Additionally, for multi-frequency noise, see as Figures 4B–D. “Amplitude” is the amplitude of the peak of the noise signals, and “Frequency” describes the noise frequencies. The number of values in “Amplitude” and “Frequency” should be the same.

After synthesizing signals, users can press the “Save” button to save the synthetic PPG to comma-separated values file (.csv), Microsoft Excel file (.xlsx), and MAT-file (.mat).

## 6. Limitations of Study and Future Work

Generating regular PPG signal using certain parameters is reproducible. If we add noise, the PPG signal cannot be reproduced as the noise addition is carried out randomly. On the other hand, generating irregular PPG signals is non-reproducible because the duration of each beat is randomly getting set. Adding noise to the generated irregular PPG signal makes it highly non-reproducible.

The next step is to generate re-entry irregular heartbeats in PPG signals, and potentially other types of abnormalities to the toolbox. The main focus of the current study was not on detecting events in irregular PPG signals with irregular heartbeats; rather, the focus was on generating irregularity in PPG signals. Another aspect of future development is to generate PPG signals with certain hemodynamic parameters (e.g., blood pressure levels) simulating the PPG templates and their associated hemodynamics parameters. This toolbox is released as version 1 (PPGSynth v1.0,) and the more templates we include the more the toolbox will be more able to generate PPG waveforms covering different irregularities (simulated cardiovascular patient groups) and noise types. One of the next steps is to generate normotensive and hypertensive PPG signals.

## 7. Summary

PPGSynth, a new publicly available toolbox, is described as a means to generate synthetic PPG waveforms. Users can easily generate a waveform across a range of sampling frequencies and can also set the length of regular and irregular PPGs. The utility can also generate specific shapes of PPGs by modifying the pulse parameter settings. These characteristics make the new toolbox useful for less experienced users that would like to generate synthetic PPGs for their research and training in physiological measurements.

## Data Availability Statement

The PPGSynth MATLAB toolbox is publicly available. The code can be downloaded via this link: https://github.com/Elgendi/PPG-Synthesis/tree/master/code and the .exe file can be downloaded via this link: https://github.com/Elgendi/PPG-Synthesis/tree/master/exe.

## Author Contributions

ME designed the study, led the investigation, drafted the manuscript for submission with revisions, and feedback from the contributing authors. QT, ZC, JA, AA, CM, RW, and ME conceived the study, provided directions, feedback, and revised the manuscript. All authors approved the final manuscript.

## Conflict of Interest

The authors declare that the research was conducted in the absence of any commercial or financial relationships that could be construed as a potential conflict of interest.
